# Expected values for the accuracy of predicted breeding values accounting for genetic differences between reference and target populations

**DOI:** 10.1186/s12711-024-00876-9

**Published:** 2024-02-29

**Authors:** Beatriz C. D. Cuyabano, Didier Boichard, Cedric Gondro

**Affiliations:** 1 INRAE, AgroParisTech, GABI, Université Paris Saclay, 78350 Jouy-en-Josas, France; 2 Department of Animal Science, Michigan State University, 474 S Shaw Ln, East Lansing, MI 48824 USA

## Abstract

**Background:**

Genetic merit, or breeding values as referred to in livestock and crop breeding programs, is one of the keys to the successful selection of animals in commercial farming systems. The developments in statistical methods during the twentieth century and single nucleotide polymorphism (SNP) chip technologies in the twenty-first century have revolutionized agricultural production, by allowing highly accurate predictions of breeding values for selection candidates at a very early age. Nonetheless, for many breeding populations, realized accuracies of predicted breeding values (PBV) remain below the theoretical maximum, even when the reference population is sufficiently large, and SNPs included in the model are in sufficient linkage disequilibrium (LD) with the quantitative trait locus (QTL). This is particularly noticeable over generations, as we observe the so-called erosion of the effects of SNPs due to recombinations, accompanied by the erosion of the accuracy of prediction. While accurately quantifying the erosion at the individual SNP level is a difficult and unresolved task, quantifying the erosion of the accuracy of prediction is a more tractable problem. In this paper, we describe a method that uses the relationship between reference and target populations to calculate expected values for the accuracies of predicted breeding values for non-phenotyped individuals accounting for erosion. The accuracy of the expected values was evaluated through simulations, and a further evaluation was performed on real data.

**Results:**

Using simulations, we empirically confirmed that our expected values for the accuracy of PBV accounting for erosion were able to correctly determine the prediction accuracy of breeding values for non-phenotyped individuals. When comparing the expected to the realized accuracies of PBV with real data, only one out of the four traits evaluated presented accuracies that were significantly higher than the expected, approaching $\sqrt{{{\text{h}}}^{2}}$.

**Conclusions:**

We defined an index of genetic correlation between reference and target populations, which summarizes the expected overall erosion due to differences in allele frequencies and LD patterns between populations. We used this correlation along with a trait’s heritability to derive expected values for the accuracy (${\text{R}}$) of PBV accounting for the erosion, and demonstrated that our derived ${\text{E}}\left[{\text{R}}|{\text{erosion}}\right]$ is a reliable metric.

**Supplementary Information:**

The online version contains supplementary material available at 10.1186/s12711-024-00876-9.

## Background

Today, many commercial livestock breeding programs use genetic merit for the selection of individuals within their programs. Genetic merits, which are often referred to as breeding values (BV), comprise the individual’s additive genetic effects that are directly transmitted to its offspring [1]. Estimation of BV relies on the relationships between individuals, and while such an estimation depends on having recorded phenotypes for the traits of interest, it is possible to predict BV for individuals without phenotypic records through their relationships with phenotyped individuals. Henderson’s mixed model equations (MME) [2–4] provided a method that yields the so-called best linear unbiased predictors (BLUP) of the individuals’ BV, a method which in its original conception used pedigree-based relationships. Combined with the rapid computational advancements during the second half of the twentieth century, Henderson’s MME (HMME) revolutionized livestock production systems, enabling large-scale genetic evaluations (i.e. the estimation of BV).

Thanks to the rapid development of molecular technologies, genotype information in the form of single nucleotide polymorphisms (SNPs) is now available at a relatively low cost for the agricultural industry. The first decade of the twenty-first century was marked by significant developments in statistical methods to perform genetic evaluation including either exclusively genomic information [5, 6], or by combining both genomic and pedigree information, either to perform the single-step genetic evaluation [7, 8], or to enhance genetic relationships even when all individuals are genotyped. These developments resulted in dramatic rates of improvement in agricultural production [9], and today BV can be obtained through either pedigree relationships, a genomic relationship matrix (GRM) [6], or the single-step relationship matrix [7, 8], by implementing Henderson’s BLUP or a variety of Bayesian methods [5, 10–14], with the reproducing kernel Hilbert spaces (RKHS) being among the most popular of the latter methods when using relationship matrices [11].

In breeding programs, obtaining predicted BV (PBV) for young candidates prior to observing their phenotypes allows the selection at a very early age, thus enabling a reduction of the generation interval, a benefit of particular relevance for example in cattle breeding populations. Thus, the accuracy of PBV is a very important factor for the success of a breeding program. However, realized accuracies of PBV remain below the theoretical maximum even when the reference population is sufficiently large, and SNPs included in the model are in sufficient linkage disequilibrium (LD) with the quantitative trait loci (QTL) within the reference population. This is particularly noticeable over generations, as we observe the so-called erosion of SNP effects [15] accompanied by the erosion of the accuracy of PBV. Erosion occurs mostly because of differences in LD patterns and allele frequencies between reference and target populations; for example, if in the reference population a SNP is in strong LD with a QTL, a large effect will be assigned to it. However, if due to segregation over generations, the LD between this SNP and the QTL becomes weaker in the target population, an effect closer to zero should be assigned to this SNP. In this paper, accuracy of the PBV will be defined as the correlation with own performance in a validation procedure, with a maximum theoretical value of $\sqrt{{{\text{h}}}^{2}}$ (where ${{\text{h}}}^{2}$ is the trait’s heritability).

The decay in prediction accuracy due to differences in allele frequencies and LD patterns, especially across generations, is a topic widely known and discussed by animal breeders and quantitative geneticists, with a number of different deterministic equations proposed [16–22]. Dekkers et al. [15] proposed a deterministic method to predict the accuracy of PBV based on selection index theory and on Fisher’s information theory, a method that depends on the effective number of chromosome segments (${{\text{M}}}_{{\text{e}}}$), which in turn relies on quantifying the erosion at the individual SNP level. While their method was successful with simulated data, for which the recombination at the individual SNP level, i.e. the erosion factor, is known, it may lead to wrong predictions of the accuracy of PBV with real datasets, for which the erosion factor is unknown and has to be estimated. In order to address this challenge in real datasets, a factor that accounts for long-distance LD was added to the deterministic formula of the predicted accuracy of PBV, however the values for this factor are quite arbitrary. Accurately quantifying the erosion at the individual SNP level is in fact, a difficult and unresolved task. It is, however, more tractable to quantify the erosion of the accuracy of the PBV through a metric based on the relationships between reference and target populations.

In this work, we propose a statistical method that accounts for erosion to derive the expected accuracy of the PBV through an index of genetic correlation (IGC) between reference and target populations. By considering the accuracy of the PBV as a population parameter measured on the target population, we evaluated our proposed approach using simulated and real data. Accurate expectations for the accuracy of PBV, accounting for erosion, will improve our understanding of the gap between the theoretical maximum, i.e. $\sqrt{{{\text{h}}}^{2}}$, and the observed prediction accuracy. Moreover, defining expectations for the accuracy of PBV based on the correlations between reference and target populations allows us to determine whether accuracies lower than $\sqrt{{{\text{h}}}^{2}}$ can be further increased by enhancing the model, or if a low accuracy is only a feature of a target population that is poorly represented by the reference population.

## Methods

### Model for the prediction of breeding values

Consider the animal model for a genetic evaluation $\mathbf{y}=\mathbf{g}+{\varvec{\upvarepsilon}}$, where $\mathbf{y}$ is a vector of the phenotypes measured in the reference population and pre-corrected for the fixed effects, $\mathbf{g}\sim N(\mathbf{0},\mathbf{W}{\upsigma }_{{\text{g}}}^{2})$ is the vector of the additive genetic effects, referred to as the BV in animal and plant breeding, ${\varvec{\upvarepsilon}}\sim N(\mathbf{0},{\mathbf{I}}_{{\text{n}}}{\upsigma }_{\upvarepsilon }^{2})$ is the vector of the random residuals, and ${\upsigma }_{{\text{g}}}^{2}$ and ${\upsigma }_{\upvarepsilon }^{2}$ are the additive genetic variance and the residual variance, respectively. $\mathbf{W}$ is the matrix of the relationship coefficients between the individuals, and we assume that this relationship can be any of the following three: (i) the pedigree-based relationship matrix, i.e. $\mathbf{W}=\mathbf{A}$; (ii) the genomic relationship matrix [6], i.e. $\mathbf{W}=\mathbf{G}$; or (iii) the single-step relationship matrix [7, 8], i.e. $\mathbf{W}=\mathbf{H}$. We emphasize here that the type of relationship (pedigree, genomic, or single-step) will not affect the future derivations and results with respect to the expected values for the accuracy of PBV.

When the goal is to predict BV for young candidates prior to observing their phenotypes, our model can be re-written as ${\mathbf{y}}_{1}=[{\mathbf{I}}_{{{\text{n}}}_{1}}{\mathbf{0}}_{{{\text{n}}}_{1}\times {{\text{n}}}_{2}}]\mathbf{g}+ {{\varvec{\upvarepsilon}}}_{1}$, such that $$\mathbf{g}=\left[\begin{array}{c}{\mathbf{g}}_{1}\\ {\mathbf{g}}_{2}\end{array}\right]\sim {\text{N}}\left(\mathbf{0},\mathbf{W}{\upsigma }_{{\text{g}}}^{2}\right)\stackrel{\scriptscriptstyle{\text{def}}}{=}{\text{N}}\left(\mathbf{0},\left[\begin{array}{cc}{\mathbf{W}}_{11}& {\mathbf{W}}_{12}\\ {\mathbf{W}}_{21}& {\mathbf{W}}_{22}\end{array}\right]{\upsigma }_{{\text{g}}}^{2}\right)$$, such that sub-index $1$ indicates the reference population of phenotyped individuals, and sub-index $2$ indicates the target population without phenotypes (young candidates). From Henderson’s MME [2–4], the analytical solutions for the breeding values ${\widehat{\mathbf{g}}}_{1}$ for the ${{\text{n}}}_{1}$ animals in the reference population, and the PBV ${\widetilde{\mathbf{g}}}_{2}$ for the ${{\text{n}}}_{2}$ animals in the target population are:

1 $${\hat{\mathbf{g}}}_{1} = {\mathbf{W}}_{11} \left( {{\mathbf{W}}_{11}\upsigma _{{\text{g}}}^{2} + {\mathbf{I}}_{{{\text{n}}_{1} }}\upsigma _{\upvarepsilon }^{2} } \right)^{ - 1} { }{\mathbf{y}}_{1}\upsigma _{{\text{g}}}^{2} ,$$

2 $${\tilde{\mathbf{g}}}_{2} = {\mathbf{W}}_{21} \left( {{\mathbf{W}}_{11}\upsigma _{{\text{g}}}^{2} + {\mathbf{I}}_{{{\text{n}}_{1} }}\upsigma _{\upvarepsilon }^{2} } \right)^{ - 1} {\mathbf{y}}_{1}\upsigma _{g}^{2} .$$

### Theoretical limit for the accuracy of predicted breeding values

Our interest lies on the accuracy of the PBV, i.e. on ${\text{R}} = \widehat{{{\text{cor}}}}\left( {{\tilde{\mathbf{g}}}_{2} ,{\mathbf{y}}_{2} } \right) = \frac{{\mathop \sum \nolimits_{{{\text{i}} = 1}}^{{{\text{n}}_{2} }} \left( {{\tilde{\text{g}}}_{{2{\text{i}}}} - {\overline{\text{g}}}_{2} } \right)\left( {{\text{y}}_{{2{\text{i}}}} - {\overline{\text{y}}}_{2} } \right)}}{{\sqrt {\mathop \sum \nolimits_{{{\text{i}} = 1}}^{{{\text{n}}_{2} }} \left( {{\tilde{\text{g}}}_{{2{\text{i}}}} - {\overline{\text{g}}}_{2} } \right)^{2} \mathop \sum \nolimits_{{{\text{i}} = 1}}^{{{\text{n}}_{2} }} \left( {{\text{y}}_{{2{\text{i}}}} - {\overline{\text{y}}}_{2} } \right)^{2} } }}$. More specifically, our interest lies on the expected value ${\text{E}}\left({\text{R}}\right)$. While the distribution of ${\text{R}}$, a realized correlation, is not straightforward, Fisher demonstrated that [23]:

3 $${\text{Z}} = {\text{log}}\left( {\frac{{1 + {\text{R}}}}{{1 - {\text{R}}}}} \right)\sim { }N\left( {{\text{log}}\left( {\frac{{1 +\uprho }}{{1 -\uprho }}} \right),\frac{4}{{{\text{n}}_{2} - 3}}} \right),$$

such that $\uprho$ is the true correlation. When predicting BV for young candidates without phenotypes, we can consider the true correlation as $\uprho ={{\text{cor}}}\left({\widehat{\mathbf{g}}}_{1}, {\mathbf{y}}_{1}\right)$, as it is intuitive that the expected prediction accuracy in the target population, *i.e.* the young candidates, is the same accuracy obtained on the reference population. If we assume that the training dataset is sufficiently large, and that the available SNPs are representative of the QTL, such that BV are accurately obtained for the reference population, we may consider the true correlation as $\uprho ={{\text{cor}}}\left({{\widehat{\mathbf{g}}}_{1},{{\mathbf{y}}_1}} \right) \approx \sqrt {{{\text{h}}^2}}$.

We acknowledge that the distributions in Eq. (3) rely on the assumption that observations are independent, an assumption that does not hold when individuals are related. However, this lack of independence among the elements in ${\mathbf{g}}_{2}$ and ${\mathbf{y}}_{2}$ has an impact only on ${\text{Var}}({\text{Z}})$. Since the interest of our present study is restricted to ${\text{E}}({\text{Z}})$, and ultimately ${\text{E}}({\text{R}})$, we will disregard potential changes to the variance of the distribution defined in Eq. (3).

**Fig. 1:**
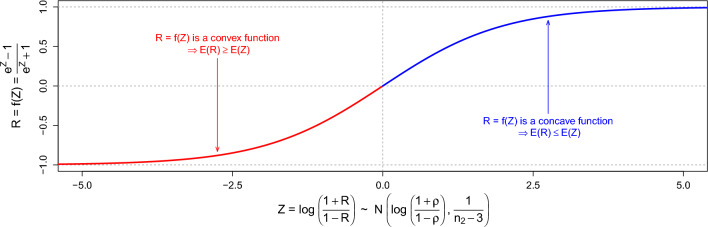
Description of the relationship between ${\text{Z}}={\text{log}}\left(\frac{1+{\text{R}}}{1-{\text{R}}}\right)$ and ${\text{R}}=\widehat{{\text{cor}}}\left({\widetilde{\mathbf{g}}}_{2},{\mathbf{y}}_{2}\right)=\frac{{{\text{e}}}^{{\text{Z}}}-1}{{{\text{e}}}^{{\text{Z}}}+1}$, highlighting the regions in which ${\text{R}}={\text{f}}({\text{Z}})$ is a convex (in red) or concave (in blue) function of $\mathrm{Z }\sim \mathrm{ N}\left({\text{log}}\left(\frac{1+\uprho }{1-\uprho }\right),\frac{4}{{{\text{n}}}_{2}-3}\right)$

The distribution of ${\text{Z}}$ will allow us to comprehend ${\text{E}}({\text{R}})$, although the exact distribution of ${\text{R}}$, a realized correlation, is not straightforward. To do so, we will study ${\text{R}}$ as a function of ${\text{Z}}$. Since ${\text{Z}}={\text{log}}\left(\frac{1+{\text{R}}}{1-{\text{R}}}\right)$, then ${\text{R}}={\text{f}}({\text{Z}})=\frac{{{\text{e}}}^{{\text{Z}}}-1}{{{\text{e}}}^{{\text{Z}}}+1}$, a function that is concave for ${\text{Z}}>0$ and convex for ${\text{Z}}<0$, as illustrated in Fig. 1. Thus, Jensen’s inequality [24] allows us to conclude that:

4 $${\text{E}}\left( {\text{R}} \right) = {\text{E}}\left[ {{\text{f}}\left( {\text{Z}} \right)} \right] \le {\text{f}}\left( {{\text{E}}\left[ {\text{Z}} \right]} \right) = \frac{{{\text{e}}^{{{\text{E}}\left( {\text{Z}} \right)}} - 1}}{{{\text{e}}^{{{\text{E}}\left( {\text{Z}} \right)}} + 1}},\quad {\text{for}}\;{\text{Z}} > 0,$$

5 $${\text{E}}\left( {\text{R}} \right) = {\text{E}}\left[ {{\text{f}}\left( {\text{Z}} \right)} \right] \ge {\text{f}}\left( {{\text{E}}\left[ {\text{Z}} \right]} \right) = \frac{{{\text{e}}^{{{\text{E}}\left( {\text{Z}} \right)}} - 1}}{{{\text{e}}^{{{\text{E}}\left( {\text{Z}} \right)}} + 1}},\quad {\text{for}}\;{\text{Z}} < 0.$$

Finally, since ${\text{E}}\left( {\text{Z}} \right) = {\text{log}}\left( {\frac{{1 +\uprho }}{{1 -\uprho }}} \right)$, as per the distribution in Eq. (3), the inequalities in Eqs. (4) and (5) can be re-written as:

6 $$\begin{aligned} {\text{E}}\left( {\text{R}} \right) \le {\text{f}}\left( {{\text{E}}\left[ {\text{Z}} \right]} \right) & = \frac{{{\text{e}}^{{{\text{E}}\left( {\text{Z}} \right)}} - 1}}{{{\text{e}}^{{{\text{E}}\left( {\text{Z}} \right)}} + 1}} = \frac{{{\text{e}}^{{{\text{log}}\left( {\frac{{1 + {\uprho }}}{{1 - {\uprho }}}} \right)}} - 1}}{{{\text{e}}^{{{\text{log}}\left( {\frac{{1 + {\uprho }}}{{1 - {\uprho }}}} \right)}} + 1}} \\ & = \frac{{\left( {\frac{{1 +\uprho }}{{1 -\uprho }}} \right) - 1}}{{\left( {\frac{{1 +\uprho }}{{1 -\uprho }}} \right) + 1}} = \frac{{\left( {1 +\uprho } \right) - \left( {1 -\uprho } \right)}}{{\left( {1 +\uprho } \right) + \left( {1 -\uprho } \right)}} \\ & = \frac{{2\uprho }}{2} =\uprho ,\quad {\text{for}}\;{\text{Z > 0,}} \\ \end{aligned}$$

7 $${\text{E}}\left( {\text{R}} \right) \ge {\uprho ,}\quad {\text{for}}\;{\text{Z < 0}}{.}$$

Because the animal model is defined as $\mathbf{y}={\mathbf{g}}+{\varvec{\upvarepsilon}}$, it is straightforward that both $\uprho = {{\text{cor}}}\left({{\widehat{\mathbf{g}}}_{1},{{\mathbf{y}}_1}} \right)$ and ${\text{R}}=\widehat{{\text{cor}}}\left({\widetilde{\mathbf{g}}}_{2},{\mathbf{y}}_{2}\right)$ should be greater than zero, and therefore we focus only on the inequality described in Eq. (6), in which all elements ${\text{Z}}$, ${\text{R}}$, and $\uprho$ assume positive values.

Some remarks must be made to conclude this section. Previous works that evaluated the accuracy of PBV traditionally assumed that ${\text{E}}\left({\text{R}}\right)\le \sqrt{{{\text{h}}}^{2}}$, while here we assume that ${\text{E}}\left({\text{R}}\right)\le\uprho = {\text{cor}}\left( {{\widehat{\mathbf{g}}}_{1},{{\bf{y}}_1}} \right)$. In fact, as previously stated, if the training dataset is sufficiently large and the QTL are correctly represented by the SNPs, the BV for the reference population are accurate enough to ensure, $\uprho \approx \sqrt{{{\text{h}}}^{2}}$. However, if the BV of the reference population are not accurate, $\uprho <\sqrt{{{\text{h}}}^{2}}$. This may occur either because of an insufficient number of samples, or because the SNPs are unable to correctly capture the QTL effects (or both). Finally, we emphasize that, in this work, we do not intend to address the drivers of inaccurate BV estimation in the reference population. Instead, we address how the genetic connections between reference and target populations impact the accuracy of the PBV for the target population of individuals without phenotypes. Therefore, ${\text{E}}\left({\text{R}}\right)\le\uprho = {\text{cor}}\left( {{\widehat{\mathbf{g}}}_{1},{{\bf{y}}_1}} \right)\le \sqrt{{{\text{h}}}^{2}}$. If the target population is well represented by the reference population, meaning that the relationships in ${\mathbf{W}}_{21}$ are strong, then ${\text{E}}\left({\text{R}}\right)$ should be in the upper boundary of the inequality in Eq. (6), reaching the equality ${\text{E}}\left({\text{R}}\right)=\uprho$. A final remark is that, if the number of records is not sufficiently large to adequately estimate ${\widehat{\mathbf{g}}}_{1}$, then ${\widehat{\mathbf{g}}}_{1}$ and ${\widehat{\mathbf{e}}}_{1}$ may present a level of correlation because of the model’s inability to adequately separate the random effects from the residual effects, resulting in an accuracy at the training population of $\uprho ={{\text{cor}}}\left({\widehat{\mathbf{g}}}_{1},{\mathbf{y}}_{1}\right)> \sqrt{{{\text{h}}}^{2}}$. In this situation, the limit for ${\text{R}}=\widehat{{\text{cor}}}\left({\widetilde{\mathbf{g}}}_{2},{\mathbf{y}}_{2}\right)$ will be $\sqrt{{{\text{h}}}^{2}}$, and thus ${\text{E}}\left({\text{R}}\right)\le\uprho ={\text{min}}\left\{{\text{cor}}\left({\widehat{\mathbf{g}}}_{1},{\mathbf{y}}_{1}\right),\sqrt{{{\text{h}}}^{2}}\right\}\le \sqrt{{{\text{h}}}^{2}}$.

### Erosion in the accuracy of predicted breeding values

In the previous section, we have established that ${\text{E}}\left({\text{R}}\right)\le\uprho ={\text{min}}\left\{{\text{cor}}\left({\widehat{\mathbf{g}}}_{1},{\mathbf{y}}_{1}\right),\sqrt{{{\text{h}}}^{2}}\right\}\le \sqrt{{{\text{h}}}^{2}}$, and that if the target population is well represented by the reference population, meaning that the relationships in ${\mathbf{W}}_{21}$ are strong, then we can expect that ${\text{E}}\left({\text{R}}\right)=\uprho$. However, if the target population is poorly represented by the reference population, the relationships in ${\mathbf{W}}_{21}$ are weak. Finally, since ${\mathbf{W}}_{21}$ is the key to obtaining the PBV (${\widetilde{\mathbf{g}}}_{2}$), as per Eq. (2), the weaker the relationships in ${\mathbf{W}}_{21}$, the more inaccurate the PBV, and we can expect that ${\text{E}}\left({\text{R}}\right)<\uprho$. The question that we address in this work is how much smaller than $\uprho$ is ${\text{E}}\left({\text{R}}\right)$.

In other words, we shall say that when ${\text{E}}\left({\text{R}}\right)<\uprho$ there is an erosion in the accuracy of PBV, and we use again Fisher’s Z-transformation [23] to quantify the eroded ${\text{E}}\left({\text{R}}\right)$. First, we must define a population parameter ${\text{r}}\in [\mathrm{0,1}]$, a single value which summarizes the relationships in ${\mathbf{W}}_{21}$, resembling a correlation. Hereafter, we will refer to ${\text{r}}$ as the index of genetic correlation (IGC) between reference and target populations, and in the next section we describe in detail how this parameter can be calculated. Note that ${\text{r}}\in [\mathrm{0,1}]$ is not simply equal to the average relationships in ${\mathbf{W}}_{21}$, and in fact obtaining ${\text{r}}$ directly from the relationship matrix $\mathbf{W}$ poses some challenges, which we address in “Index of genetic correlation between populations” section, along with two suggested methods to estimate ${\text{r}}$.

**Fig. 2:**
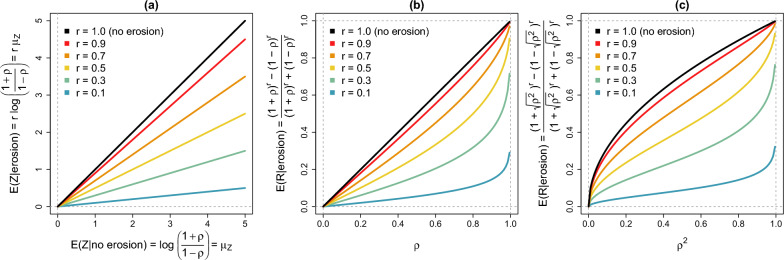
Description of ${\text{E}}({\text{R}}|{\text{erosion}})=\frac{{\left(1+\uprho \right)}^{{\text{r}}}-{\left(1-\uprho \right)}^{{\text{r}}}}{{\left(1+\uprho \right)}^{{\text{r}}}+{\left(1-\uprho \right)}^{{\text{r}}}}$ as a function of (**a)** ${\text{E}}\left({\text{Z}}|\mathrm{no\hspace{0.1cm}erosion}\right)={\text{log}}\left(\frac{1+\uprho }{1-\uprho }\right)={\upmu }_{{\text{Z}}}$; (**b)** $\uprho ={\text{cor}}\left({\widehat{\mathbf{g}}}_{1},{\mathbf{y}}_{1}\right)$; (**c)** ${\uprho }^{2}={{\text{cor}}}^{2}\left({\widehat{\mathbf{g}}}_{1},{\mathbf{y}}_{1}\right)$

Returning to the Z-transformed accuracy of PBV, we have that ${\text{E}}\left({\text{Z}}\right)={\text{log}}\left(\frac{1+\uprho }{1-\uprho }\right)$, as per the distribution in Eq. (3), and we will say that this expected value holds when there is no erosion effect, i.e. ${\text{E}}\left({\text{Z}}|\mathrm{no\hspace{0.1cm}erosion}\right)={\text{log}}\left(\frac{1+\uprho }{1-\uprho }\right)={\upmu }_{{\text{Z}}}$. We hypothesized that the eroded ${\text{E}}\left({\text{Z}}\right)$ is linearly affected by the IGC (${\text{r}}$), thus:

8 $${\text{E}}\left( {{\text{Z}}|{\text{erosion}}} \right) = {\text{r log}}\left( {\frac{{1 +\uprho }}{{1 -\uprho }}} \right) = {\text{r}}\upmu _{{\text{Z}}} ,$$

such that ${\text{E}}\left({\text{Z}}|{\text{erosion}}\right)\underset{\mathrm{ r}\to 1 }{\to }{\text{E}}\left({\text{Z}}|\mathrm{no\hspace{0.1cm}erosion}\right)$, and ${\text{E}}\left({\text{Z}}|{\text{erosion}}\right)\underset{\mathrm{ r}\to 0 }{\to }0$, this last scenario being equivalent to a reference population, thus distinct from the target population, this BV cannot be predicted at all. Finally, operating with ${\text{R}}={\text{f}}({\text{Z}})=\frac{{{\text{e}}}^{{\text{Z}}}-1}{{{\text{e}}}^{{\text{Z}}}+1}$, we have:

9 $$\begin{aligned} {\text{E}}\left( {{\text{R}}|{\text{erosion}}} \right) & = {\text{f}}\left( {{\text{E}}\left[ {{\text{Z}}|{\text{erosion}}} \right]} \right) = \frac{{{\text{e}}^{{{\text{E}}\left( {{\text{Z}}|{\text{erosion}}} \right)}} - 1}}{{{\text{e}}^{{{\text{E}}\left( {{\text{Z}}|{\text{erosion}}} \right)}} + 1}} \\ & = \frac{{{\text{e}}^{{{\text{r log}}\left( {\frac{{1 +\uprho }}{{1 -\uprho }}} \right)}} - 1}}{{{\text{e}}^{{{\text{r log}}\left( {\frac{{1 +\uprho }}{{1 -\uprho }}} \right)}} + 1}} = \frac{{{\text{e}}^{{{\text{log}}\left( {\frac{{1 +\uprho }}{{1 -\uprho }}} \right)^{{\text{r}}} }} - 1}}{{{\text{e}}^{{{\text{log}}\left( {\frac{{1 +\uprho }}{{1 -\uprho }}} \right)^{{\text{r}}} }} + 1}} \\ & = \frac{{\left( {\frac{{1 +\uprho }}{{1 -\uprho }}} \right)^{{\text{r}}} - 1}}{{\left( {\frac{{1 +\uprho }}{{1 -\uprho }}} \right)^{{\text{r}}} + 1}} = \frac{{\left( {1 +\uprho } \right)^{{\text{r}}} - \left( {1 -\uprho } \right)^{{\text{r}}} }}{{\left( {1 +\uprho } \right)^{{\text{r}}} + \left( {1 -\uprho } \right)^{{\text{r}}} }}, \\ \end{aligned}$$

such that ${\text{E}}\left({\text{R}}|{\text{erosion}}\right)\underset{\mathrm{ r}\to 1 }{\to }\uprho ={\text{E}}\left({\text{R}}|\mathrm{no\hspace{0.1cm}erosion}\right)$, and ${\text{E}}\left({\text{R}}|{\text{erosion}}\right)\underset{\mathrm{ r}\to 0 }{\to }0$. Figure 2 describes the behaviour of ${\text{E}}({\text{R}}|{\text{erosion}})=\frac{{\left(1+\uprho \right)}^{{\text{r}}}-{\left(1-\uprho \right)}^{{\text{r}}}}{{\left(1+\uprho \right)}^{{\text{r}}}+{\left(1-\uprho \right)}^{{\text{r}}}}$ as a function of ${\text{E}}\left({\text{Z}}|\mathrm{no\hspace{0.1cm}erosion}\right)={\upmu }_{{\text{Z}}}$, $\uprho$, and ${\uprho }^{2}$, and shows that ${\text{E}}({\text{R}}|{\text{erosion}})$ is an increasing function on both $\uprho$ and ${\text{r}}$. The description of ${\text{E}}({\text{R}}|{\text{erosion}})$ as a function of ${\uprho }^{2}$, although redundant with the description of ${\text{E}}({\text{R}}|{\text{erosion}})$ as a function of $\uprho$, has relevance to interpret ${\text{E}}({\text{R}}|{\text{erosion}})$ on the same scale as a function of the trait’s heritability (${{\text{h}}}^{2}$).

### Index of genetic correlation between populations

In order to quantify ${\text{E}}({\text{R}}|{\text{erosion}})=\frac{{\left(1+\uprho \right)}^{{\text{r}}}-{\left(1-\uprho \right)}^{{\text{r}}}}{{\left(1+\uprho \right)}^{{\text{r}}}+{\left(1-\uprho \right)}^{{\text{r}}}}$, the IGC represented by ${\text{r}}$ must be calculated. As mentioned in the previous section, obtaining ${\text{r}}$ directly from the relationship matrix $\mathbf{W}$ can be challenging. In the next paragraph, we show how to calculate ${\text{r}}$ when $\mathbf{W}=\mathbf{G}$, the genomic relationship matrix. This method is computationally heavy and may be unfeasible for very large datasets. We also propose a simpler method which uses empirical results on simulated phenotypes and can be a practical alternative that may be applied to any type of relationship matrix $\mathbf{W}$ (pedigree, genomic or single-step). A sample code for implementing both methods to calculate the IGC in R is provided in Additional file 1.

#### IGC calculated from genomic data

Let $\mathbf{M}$ be a ${\text{n}}\times {\text{m}}$ centered and scaled matrix of SNP-genotypes, where the scaling factor is ${\left({\sum }_{{\text{j}}=1}^{{\text{m}}}2{{\text{p}}}_{{\text{j}}}(1-{{\text{p}}}_{{\text{j}}})\right)}^{-1/2}$ and ${{\text{p}}}_{{\text{j}}}^{\prime}{\text{s}}$ are the alleles frequencies, and its singular-value decomposition (SVD) is $\mathbf{M}=\mathbf{U}\mathbf{D}\mathbf{V}^{\prime}$. In this SVD, $\mathbf{D}=\mathbf{d}\mathbf{i}\mathbf{a}\mathbf{g}({{\text{d}}}_{1},{{\text{d}}}_{2},\dots ,{{\text{d}}}_{{\text{n}}-1} ,{{\text{d}}}_{{\text{n}}})$ is a diagonal matrix of the ${\text{n}}$ singular-values, such that ${{\text{d}}}_{1}\ge \dots \ge {{\text{d}}}_{{\text{n}}}\ge 0$ with ${{\text{d}}}_{{\text{i}}}=0$ for every ${\text{i}}>{\text{rank}}(\mathbf{M})$; ${\mathbf{U}}_{{\text{n}}\times {\text{n}}}=[{\mathbf{U}}_{1}\dots {\mathbf{U}}_{{\text{n}}}]$ and ${\mathbf{V}}_{{\text{m}}\times {\text{n}}}=[{\mathbf{V}}_{1}\dots {\mathbf{V}}_{{\text{n}}}]$ are matrices of unitary eigen-vectors, such that ${\mathbf{U}}^{\prime} \mathbf{U}=\mathbf{U}{\mathbf{U}}^{\prime}={\mathbf{I}}_{{\text{n}}}$ and ${\mathbf{V}}^{\prime}\mathbf{V}={\mathbf{I}}_{{\text{n}}}$.

Each of the components ${{\text{d}}}_{1}^{2},\dots ,{{\text{d}}}_{{\text{n}}}^{2}$ explains a portion of the variation from the whole system $\mathbf{M}$; each element ${{\text{U}}}_{{\text{ik}}}$(${\text{i}}=1,\dots ,{\text{n}}$) in ${\mathbf{U}}_{{\text{k}}}=[{{\text{U}}}_{1{\text{k}}}\dots {{\text{U}}}_{{\text{nk}}}]^{\prime}$ represents the contribution of individual ${\text{i}}$ to the variation explained by component ${\text{k}}$; each element ${{\text{V}}}_{{\text{jk}}}$(${\text{j}}=1,\dots ,{\text{m}}$) in ${\mathbf{V}}_{{\text{k}}}=[{{\text{V}}}_{1{\text{k}}}\dots {{\text{V}}}_{{\text{mk}}}]^{\prime}$ represents the contribution of SNP ${\text{j}}$ to the variation explained by component ${\text{k}}$.

**Fig. 3:**
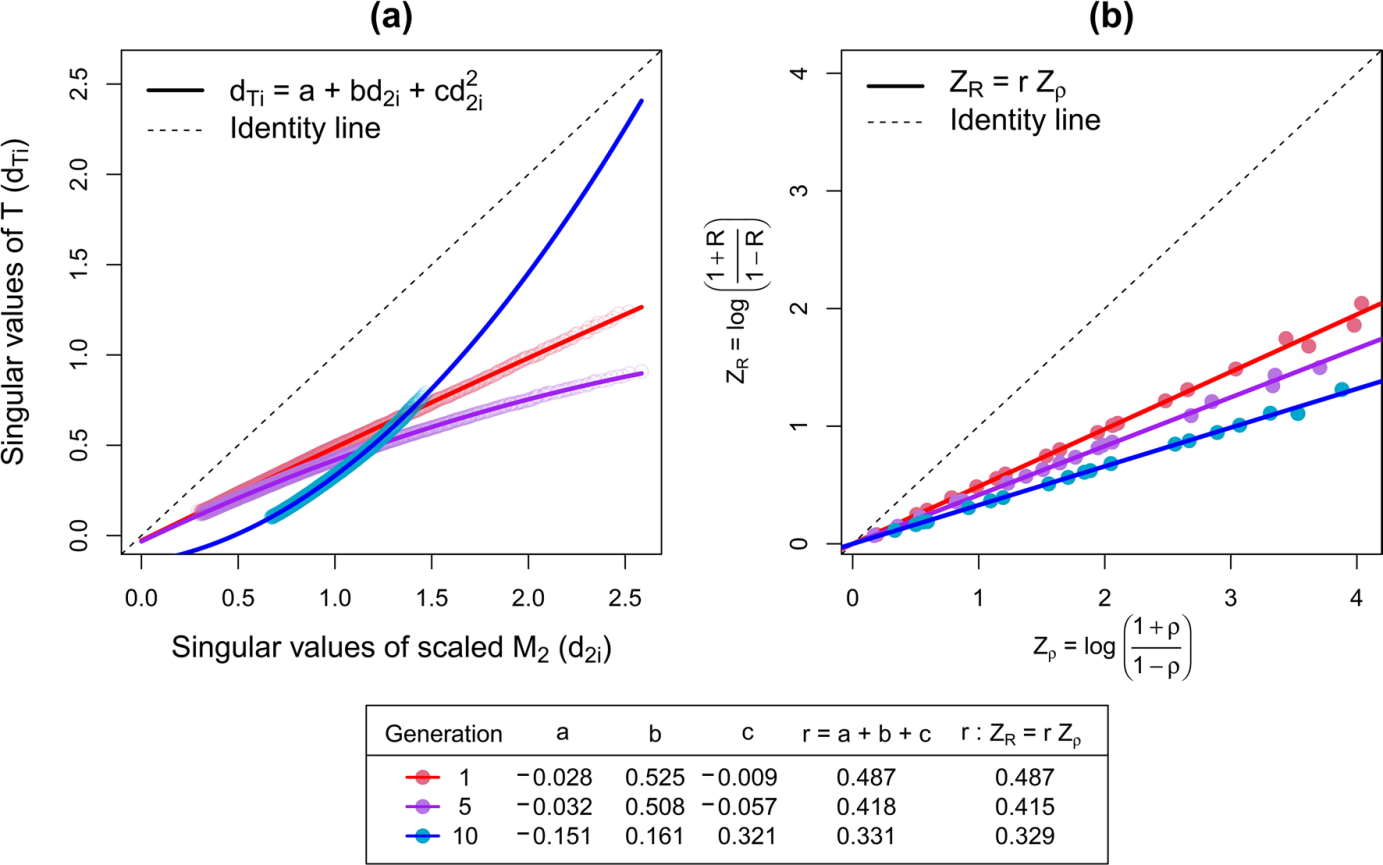
Index of genetic correlation between the simulated reference and three different target populations (one, five, and ten generations after the base reference population) using (**a**) singular-value decompositions on the matrices of SNP-genotypes as proposed in section IGC calculated from genomic data (${\text{r}}={\text{a}}+{\text{b}}+{\text{c}}$); (**b)** simulated phenotypes and their breeding values solutions as proposed in section IGC calculated from simulated phenotypes (${\text{log}}\left(\frac{1+{\text{R}}}{1-{\text{R}}}\right)={\text{rlog}}\left(\frac{1+\uprho }{1-\uprho }\right)$)

To obtain the IGC between reference and target populations (${\text{r}}$), we need to compare the different contributions of the SNPs to the system’s variation in the two populations. To do so, we perform the aforementioned SVD on ${\mathbf{M}}_{1}$ and ${\mathbf{M}}_{2}$ (bearing in mind that sub-index $1$ refers to the reference population and sub-index $2$ refers to the target population), then build a matrix $\mathbf{T}=\sqrt{{({\text{n}}}_{2}/{{\text{n}}}_{1})}{{\mathbf{V}}_{2}^{\prime}\mathbf{V}}_{1}{\mathbf{D}}_{1}$ which correlates the contributions of the SNPs in both populations, while correcting for the different population sizes, and weighting these correlations by the singular-values of the reference population. Note that the term ${{\mathbf{V}}_{2}^{\prime}\mathbf{V}}_{1}{\mathbf{D}}_{1}$ that is used to define matrix $\mathbf{T}$ is not arbitrary; this term is the kernel of the solution to ${\widetilde{\mathbf{g}}}_{2}$ in Eq. (2), when ${\mathbf{W}}_{21}={\mathbf{M}}_{2}^{\prime}{\mathbf{M}}_{1}$ and ${\mathbf{W}}_{11}={\mathbf{M}}_{1}{\mathbf{M}}_{1}^{\prime}$, and we replace ${\mathbf{M}}_{2}$ and ${\mathbf{M}}_{1}$ by their SVD. By saying that ${{\mathbf{V}}_{2}^{\prime}\mathbf{V}}_{1}{\mathbf{D}}_{1}$ is the kernel of the solution to ${\widetilde{\mathbf{g}}}_{2}$ we mean that for any trait with the same reference and target populations, ${{\mathbf{V}}_{2}^{\prime}\mathbf{V}}_{1}{\mathbf{D}}_{1}$ is a systematic linear transformation on the observed phenotypes that dictates the projected solutions ${\widetilde{\mathbf{g}}}_{2}$ for any set of observed performances, and for any heritability. Next, we obtain the SVD $\mathbf{T}={\mathbf{U}}_{{\text{T}}}{\mathbf{D}}_{{\text{T}}}{\mathbf{V}}_{{\text{T}}}^{\prime}$, and perform the linear regression ${\mathbf{D}}_{{\text{T}}}\sim {\mathbf{D}}_{2}$ with a quadratic term, i.e., we fit ${{\text{d}}}_{{\text{Ti}}}={{\text{a}}+{\text{bd}}}_{2{\text{i}}}+{{\text{cd}}}_{2{\text{i}}}^{2}$. Finally, based on extensive observational testing on empirical results, the IGC between reference and target populations can be calculated as $\mathrm{r }=\mathrm{ a}+{\text{b}}+{\text{c}}$. Figure 3a presents an example of the ${\mathbf{D}}_{{\text{T}}}\sim {\mathbf{D}}_{2}$ obtained for different scenarios of relationships between reference and target populations, which were consistently repeating the pattern of a linear or quadratic relationship between ${{\text{d}}}_{{\text{Ti}}}\sim {{\text{d}}}_{2{\text{i}}}$. Throughout all the simulated replicates, we observed that the sum of the coefficients ${\text{a}}+{\text{b}}+{\text{c}}$ was always between 0 and 1, which led us to attempt setting $\mathrm{r }=\mathrm{ a}+{\text{b}}+{\text{c}}$, and finally observing that this value empirically satisfied ${\text{E}}({\text{R}}|{\text{erosion}})=\frac{{\left(1+\uprho \right)}^{{\text{r}}}-{\left(1-\uprho \right)}^{{\text{r}}}}{{\left(1+\uprho \right)}^{{\text{r}}}+{\left(1-\uprho \right)}^{{\text{r}}}}$ for the replicates.

#### IGC calculated from simulated phenotypes

A less computationally demanding alternative to the method proposed to obtain the IGC ${\text{r}}$ is to use simulated phenotypes. There are two advantages from simulating phenotypes: (1) there is no computational burden from performing SVD on the genotype matrices; and (2) it enables obtaining ${\text{r}}$ irrespective of the genotypes being available.

The first step is to define an arbitrary phenotypic variance ${\upsigma }_{{\text{y}}}^{2}$, and then simulate $\mathbf{g}\sim N\left(\mathbf{0},\mathbf{W}{{{\text{h}}}^{2}}\upsigma _{{\text{y}}}^{2}\right)$ and ${\varvec{\upvarepsilon}}\sim N\left(\mathbf{0},{\mathbf{I}}_{{\mathbf{n}}_{1}+{\mathbf{n}}_{2}}{(1-{{\text{h}}}^{2}) }\upsigma_{\text{y}}^{2}\right)$ for a sequence of ${{\text{h}}}^{2}$ that covers a range from low to high heritabilities. If genotypes are available for all animals, $\mathbf{g}$ can be simulated from the genotypes instead, by simulating the vector of SNP effects ${\varvec{\upalpha}}\sim N\left(\mathbf{0},{\mathbf{I}}_{{\text{m}}}\frac{{{{\text{h}}}^{2} }\upsigma_{{\text{y}}}^{2}}{{\sum }_{{\text{j}}=1}^{{\text{m}}}2{{\text{p}}}_{{\text{j}}}(1-{{\text{p}}}_{{\text{j}}})}\right)$ and setting $\mathbf{g}=\mathbf{M}{\varvec{\upalpha}}$, such that $\mathbf{M}$ is the (centred) matrix of SNP-genotypes (${\text{n}}\times {\text{m}}$). The simulated phenotypes are then $\mathbf{y}=\mathbf{g}+{\varvec{\upvarepsilon}}$.

The next step is to obtain, for each ${{\text{h}}}^{2}$, the BV for the reference and the PBV for the target populations as established in Eqs. (1) and (2), setting ${\upsigma }_{{\text{g}}}^{2}={{{\text{h}}}^{2} }\upsigma_{{\text{y}}}^{2}$, i.e. ${\widehat{\mathbf{g}}}_{1}={\mathbf{W}}_{11}{\left({\mathbf{W}}_{11}{\upsigma }_{{\text{g}}}^{2}+{\mathbf{I}}_{{\text{n}}}{\upsigma }_{\upvarepsilon }^{2}\right)}^{-1} {\mathbf{y}}_{1}{{{\text{h}}}^{2} }\upsigma_{{\text{y}}}^{2}$ and ${\widetilde{\mathbf{g}}}_{2}={\mathbf{W}}_{21}{\left({\mathbf{W}}_{11}{\upsigma }_{{\text{g}}}^{2}+{\mathbf{I}}_{{\text{n}}}{\upsigma }_{\upvarepsilon }^{2}\right)}^{-1} {\mathbf{y}}_{1}{{{\text{h}}}^{2} }\upsigma_{{\text{y}}}^{2}$, and to obtain $\hat {\uprho} =\widehat{{\text{cor}}}\left({\widehat{\mathbf{g}}}_{1},{\mathbf{y}}_{1}\right)$ and ${\text{R}}=\widehat{{\text{cor}}}\left({\widetilde{\mathbf{g}}}_{2},{\mathbf{y}}_{2}\right)$. By doing this procedure for each ${{\text{h}}}^{2}$, a sample of $\uprho$’s and ${\text{R}}$’s is generated. Now the Z-transformed correlations are calculated: ${{\text{Z}}}_{\uprho }={\text{log}}\left(\frac{1+\uprho }{1-\uprho }\right)$ and ${{\text{Z}}}_{{\text{R}}}={\text{log}}\left(\frac{1+{\text{R}}}{1-{\text{R}}}\right)$.

Finally, the last step is to perform the linear regression ${{\text{Z}}}_{{\text{R}}}\sim {{\text{Z}}}_{\uprho }$ without an intercept, and according to Eq. (8), the slope of this regression is the IGC, i.e. ${\text{r}}=\frac{\widehat{{\text{cov}}}({{\text{Z}}}_{{\text{R}}},{{\text{Z}}}_{\uprho })}{\widehat{{\text{Var}}}({{\text{Z}}}_{\uprho })}$.

### Data for the empirical study

#### Simulated data

We used the R package GenEval (https://github.com/bcuyabano/GenEval) to simulate 50 k SNPs and additive phenotypes (${\upsigma }_{{\text{y}}}^{2}=100$) for a wide range of heritabilities ${{\text{h}}}^{2}$ = 0.05, 0.15,…, 0.9, 0.95, using a random subset of 2 k SNPs as QTL. SNP-genotypes were simulated in LD, as per the function simGeno() from the R package GenEval, with the LD structure set to resemble that of a cattle population. A base reference population of 5000 individuals was used to estimate variance components using the residual maximum likelihood (REML) [25, 26] and then to obtain the PBV as in Eq. (2), for three different target populations (1000 individuals each) with an increasing number of generations (one, five, and ten) from the base reference population. All the generations were simulated with a 50/50 ratio of males and females, and random mating was performed with no selection. For each ${{\text{h}}}^{2}$ = 0.05, 0.15,…, 0.9, 0.95, we simulated 500 independent replicates of phenotypes for the entire population (base and generations one, five, and ten), thus creating a large sample of ${\widehat{{\text{h}}}}^{2}$, $\widehat{\uprho }={\text{min}}\left\{\widehat{{\text{cor}}}\left({\widehat{\mathbf{g}}}_{1},{\mathbf{y}}_{1}\right),\sqrt{{\widehat{{\text{h}}}}^{2}}\right\}$, and ${\text{R}}=\widehat{{\text{cor}}}\left({\widetilde{\mathbf{g}}}_{2},{\mathbf{y}}_{2}\right)$ at each heritability level.

We performed this study for $\mathbf{W}=\mathbf{G}$, which allowed us to calculate the IGC ${\text{r}}$ with both proposed methods, and to compare the values obtained. We wanted to ensure $\widehat{\uprho }={\text{min}}\left\{\widehat{{\text{cor}}}\left({\widehat{\mathbf{g}}}_{1},{\mathbf{y}}_{1}\right),\sqrt{{\widehat{{\text{h}}}}^{2}}\right\}\approx \sqrt{{\widehat{{\text{h}}}}^{2}}$, thus the QTL were kept among the genotypes used for analysis. Then, we compared the realized prediction accuracies ${\text{R}}=\widehat{{\text{cor}}}\left({\widetilde{\mathbf{g}}}_{2},{\mathbf{y}}_{2}\right)=\frac{{\sum }_{{\text{i}}=1}^{{{\text{n}}}_{2}}({\widetilde{{\text{g}}}}_{2{\text{i}}}-{\overline{{\text{g}}} }_{2})({{\text{y}}}_{2{\text{i}}}-{\overline{{\text{y}}} }_{2})}{\sqrt{{\sum }_{{\text{i}}=1}^{{{\text{n}}}_{2}}{\left({\widetilde{{\text{g}}}}_{2{\text{i}}}-{\overline{{\text{g}}} }_{2}\right)}^{2}{\sum }_{{\text{i}}=1}^{{{\text{n}}}_{2}}{\left({{\text{y}}}_{2{\text{i}}}-{\overline{{\text{y}}} }_{2}\right)}^{2}}}$ with the theoretical curve ${\text{E}}({\text{R}}|{\text{erosion}})=\frac{{\left(1+\uprho \right)}^{{\text{r}}}-{\left(1-\uprho \right)}^{{\text{r}}}}{{\left(1+\uprho \right)}^{{\text{r}}}+{\left(1-\uprho \right)}^{{\text{r}}}}$, to evaluate how accurately this equation quantifies the erosion in the accuracy of PBG. The comparison of ${\text{R}}$ to the theoretical curve $\frac{{\left(1+\uprho \right)}^{{\text{r}}}-{\left(1-\uprho \right)}^{{\text{r}}}}{{\left(1+\uprho \right)}^{{\text{r}}}+{\left(1-\uprho \right)}^{{\text{r}}}}$ was performed for both $\uprho =\sqrt{{\widehat{{\text{h}}}}^{2}}$ and $\uprho ={\text{min}}\left\{\widehat{{\text{cor}}}\left({\widehat{\mathbf{g}}}_{1},{\mathbf{y}}_{1}\right),\sqrt{{\widehat{{\text{h}}}}^{2}}\right\}$, to test our hypothesis that the latter is a more suitable measure to be considered, even in a scenario in which $\widehat{{\text{cor}}}\left({\widehat{\mathbf{g}}}_{1},{\mathbf{y}}_{1}\right)\approx \sqrt{{\widehat{{\text{h}}}}^{2}}$.

#### Real data

We compared the accuracies of PBV to their derived expected values using real data from a dairy cattle population within a breeding program. In total, 9636 cows were used as the reference population, and the target population comprised 2130 cows. The pedigree relationship matrix $\mathbf{A}$ was built by tracing back three generations on the pedigree for each of the 11,766 cows used for the analysis. These 11,766 animals were a subset of an original dataset comprising records from ~ 100,000 cows covering six generations within the breeding program, i.e. this is a population under selection. Our subset ensured that all cows in the target population had their dams in the reference population, and that all dams could be fully traced back by three generations in the pedigree. The subset of animals belonged to the last three generations of the breeding program.

The phenotypes were available for the cows in the form of yield deviations (YD), and four traits were evaluated: one fertility (FERT), one health (HEALTH), and two production (PROD1, PROD2) traits. A preliminary study indicated that the heritabilities of these traits were ${{\text{h}}}_{{\text{FERT}}}^{2}=0.02$, ${{\text{h}}}_{{\text{HEALTH}}}^{2}=0.188$, ${{\text{h}}}_{{\text{PROD}}1}^{2}=0.375$, and ${{\text{h}}}_{{\text{PROD}}2}^{2}=0.625$.

Both pedigree information and 53,469 autosomal SNP-genotypes (EuroGMD v1, a customized ILLUMINA genotyping microarray that contains approximately 70,000 SNPs) were available for all animals, allowing us to perform the study with the three possible genetic relationship matrices: $\mathbf{W}=\mathbf{A}$ (pedigree), $\mathbf{W}=\mathbf{G}$ (genomic), and $\mathbf{W}=\mathbf{H}$ (single-step, using genotypes for all animals in the target population and for 25% of the animals in the reference population). For this study on real data, we applied only the method that uses simulated phenotypes to obtain the IGC (${\text{r}}$), and these additive phenotypes (${\upsigma }_{{\text{y}}}^{2}=100$) were simulated for heritabilities ${{\text{h}}}^{2}$ = 0.1, 0.2,…, 0.9, using a random subset of 2 k SNPs as QTL. Finally, the expected prediction accuracies accounting for erosion were calculated for each studied trait, and their values compared to the accuracies obtained with real phenotypes (in the form of the yield deviations, as mentioned previously), *i.e.* $R=\widehat{{\text{cor}}}\left({\widetilde{\mathbf{g}}}_{2},{\mathbf{y}}_{2}\right)$.

### Genomic BLUP reference for relationships between populations and reliability of prediction

We compared our derived expected accuracy of prediction ${\text{E}}({\text{R}}|{\text{erosion}})=\frac{{\left(1+\uprho \right)}^{{\text{r}}}-{\left(1-\uprho \right)}^{{\text{r}}}}{{\left(1+\uprho \right)}^{{\text{r}}}+{\left(1-\uprho \right)}^{{\text{r}}}}$ to the theoretical mean reliability obtained from Henderson’s MME [2–4], assuming the same animal model that was considered to compute ${\text{E}}({\text{R}}|{\text{erosion}})$, i.e. $\mathbf{y}=\mathbf{g}+{\varvec{\upvarepsilon}}$, with $\mathbf{y}$ being the vector of the phenotypes measured in the reference population and pre-corrected for the fixed effects. The theoretical mean reliability of the genomic (G)BLUP can be considered as the following average reliability in the target population:

10 $${\text{E}}({\text{R}}|{\text{GBLUP}}) = \frac{{\sqrt {{\hat{\text{h}}}^{2} } }}{{{\text{n}}_{2} }}\mathop \sum \limits_{{{\text{i}} = 1}}^{{{\text{n}}_{2} }} \sqrt {\left[ {{\mathbf{W}}_{21} {\mathbf{W}}_{11}^{ - 1} \left( {{\mathbf{W}}_{11} - \left( {{\mathbf{LHS}} \times {\hat{\upsigma }}_{{\text{g}}}^{2} } \right)^{ - 1} } \right){\mathbf{W}}_{11}^{ - 1} {\mathbf{W}}_{12} } \right]_{{{\text{ii}}}} } ,$$

such that $\mathbf{L}\mathbf{H}\mathbf{S}$ is the left-hand-side of Henderson’s MME for BLUP, and the sub-index $ii$ indicates the diagonal elements of the matrix inside the square brackets.

Moreover, in order to assess our proposed IGC (${\text{r}}$), we compared its value to the average weighted relationships between reference and target populations:

11 $${\text{r}}_{{{\text{GBLUP}}}} = \frac{1}{{{\text{n}}_{2} }}\mathop \sum \limits_{{{\text{i}} = 1}}^{{{\text{n}}_{2} }} \sqrt {\left[ {{\mathbf{W}}_{21} {\text{W}}_{11}^{ - 1} {\mathbf{W}}_{12} } \right]_{{{\text{ii}}}} } .$$

These comparisons were performed on the simulated data only, as their purpose was to verify if our derived expected value ${\text{E}}({\text{R}}|{\text{erosion}})$ and IGC (${\text{r}}$) were a better fit for making inferences on the expected accuracy of prediction.

## Results

Figure 3 presents the IGC between the reference and the three different target populations (one, five, and ten generations after the base reference population) using the two proposed methods. Figure 3a presents the relationship between the singular values of $\mathbf{T}=\sqrt{{({\text{n}}}_{2}/{{\text{n}}}_{1})}{{\mathbf{V}}_{2}^{\mathrm{^{\prime}}}\mathbf{V}}_{1}{\mathbf{D}}_{1}$ (${{\text{d}}}_{{\text{Ti}}}$) and the singular values of the scaled ${\mathbf{M}}_{2}$ (${{\text{d}}}_{2{\text{i}}}$), used to obtain the IGC calculated from genomic data in which ${\text{r}}={\text{a}}+{\text{b}}+{\text{c}}$, such that ${{\text{d}}}_{{\text{Ti}}}={{\text{a}}+{\text{bd}}}_{2{\text{i}}}+{{\text{cd}}}_{2{\text{i}}}^{2}$, as described in the Methods section. Figure 3b presents the linear relationship between ${{\text{Z}}}_{{\text{R}}}$ and ${{\text{Z}}}_{\uprho }$, used to obtain the IGC calculated from simulated phenotypes, in which ${\text{r}}$ satisfies ${{\text{Z}}}_{{\text{R}}}=r{{\text{Z}}}_{\uprho }$. We observed that the values of ${\text{r}}$ obtained with the two proposed methods are very similar for the three target populations. We observed that ${\text{r}}<1$ for all target populations, and as expected, it decreases when the number of generations of the target population from the base reference population increases. The results presented for ${\text{r}}$ obtained using the simulated phenotypes in Fig. 3b were based on one of the 500 replicates. However, when analysing ${\text{r}}$ for each one of those replicates, the values are comparable with standard deviations of 0.01, 0.005, and 0.005, respectively, for the target populations one, five, and ten generations apart from the base reference population.

**Fig. 4:**
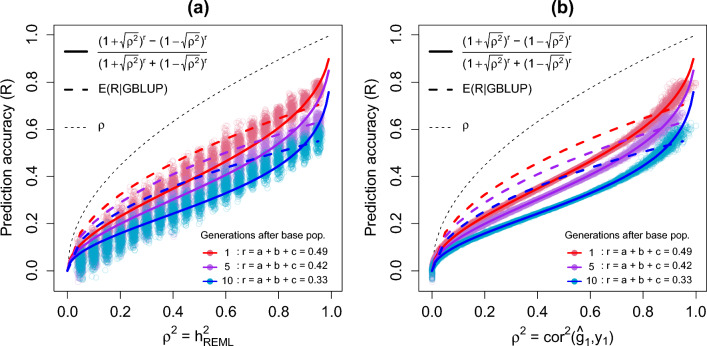
Relationship between the realized prediction accuracy ${\text{R}}=\widehat{{\text{cor}}}\left({\widetilde{\mathbf{g}}}_{2},{\mathbf{y}}_{2}\right)$ and (**a)** the REML heritability estimates ${\widehat{{\text{h}}}}^{2}$; (**b)** the squared accuracy of the breeding values of the reference population, ${\uprho }^{2}={\widehat{{\text{cor}}}}^{2}\left({\widehat{\mathbf{g}}}_{1},{\mathbf{y}}_{1}\right)$. Results presented are of the 500 replicates of simulated phenotypes for each ${{\text{h}}}^{2}$

Figure 4 presents the relationship between the realized prediction accuracy ${\text{R}}=\widehat{{\text{cor}}}\left({\widetilde{\mathbf{g}}}_{2},{\mathbf{y}}_{2}\right)$ and both the REML heritability estimates ${\widehat{{\text{h}}}}^{2}$ and the squared accuracy of the breeding values of the reference population, ${\widehat{{\text{cor}}}}^{2}\left({\widehat{\mathbf{g}}}_{1},{\mathbf{y}}_{1}\right)\approx {\widehat{{\text{h}}}}^{2}$. We observed that there is a greater variation of ${\text{R}}$ with respect to the heritability estimates, than with respect to the accuracy of the BV in the reference population. Since for all three target populations ${\text{r}}<1$, we expected that all ${\text{R}}<\sqrt{{\widehat{{\text{h}}}}^{2}}$ and ${\text{R}}<\uprho$, and this was confirmed by the results presented in Fig. 4a and b. Then, we evaluated how well the theoretical curve $\frac{{\left(1+\uprho \right)}^{{\text{r}}}-{\left(1-\uprho \right)}^{{\text{r}}}}{{\left(1+\uprho \right)}^{{\text{r}}}+{\left(1-\uprho \right)}^{{\text{r}}}}$ described the observed results, and compared it to the average GBLUP reliability in the target population, calculated as in Eq. (10). In Fig. 4a, we observed that the theoretical curve is a reasonable mean to describe the relationship between ${\text{R}}$ and ${\uprho }^{2}= {\widehat{{\text{h}}}}^{2}$, however it overestimates ${\text{R}}$ for the very low or very high heritabilities. This is not surprising, since in fact, the issue with a very lowly or very highly heritable trait is that, due to heritabilities being close to the boundaries of possible values, we expect a loss in the accuracy of BV for the reference population, i.e. $\widehat{{\text{cor}}}\left({\widehat{\mathbf{g}}}_{1},{\mathbf{y}}_{1}\right)<\sqrt{{\widehat{{\text{h}}}}^{2}}$. This result supports our hypothesis that the theoretical curve $\frac{{\left(1+\uprho \right)}^{{\text{r}}}-{\left(1-\uprho \right)}^{{\text{r}}}}{{\left(1+\uprho \right)}^{{\text{r}}}+{\left(1-\uprho \right)}^{{\text{r}}}}$ will better describe the relationship between ${\text{R}}$ and ${\uprho }^{2}= {\widehat{{\text{cor}}}}^{2}\left({\widehat{\mathbf{g}}}_{1},{\mathbf{y}}_{1}\right)$, presented in Fig. 4b, in which we observed that the theoretical curves are very accurate to describe this relationship. In both panels (a) and (b) of Fig. 4, we observed that the average GBLUP reliability in the target population failed to correctly describe the average realized prediction accuracy ${\text{R}}=\widehat{{\text{cor}}}\left({\widetilde{\mathbf{g}}}_{2},{\mathbf{y}}_{2}\right)$ across the range of heritabilities. For the simulations with ${{\text{h}}}^{2}$ below 0.8, ${\text{E}}\left({\text{R}}|{\text{GBLUP}}\right)$ does serve as an upper boundary, closer to the realized results when ${\text{R}}$ is compared to ${\uprho }^{2}= \sqrt{{\widehat{{\text{h}}}}^{2}}$ in Fig. 4a, than when ${\text{R}}$ is compared to ${\uprho }^{2}= {\widehat{{\text{cor}}}}^{2}\left({\widehat{\mathbf{g}}}_{1},{\mathbf{y}}_{1}\right)$ in Fig. 4b. For the simulations with ${{\text{h}}}^{2}$ above 0.8, ${\text{E}}\left({\text{R}}|{\text{GBLUP}}\right)$ was much closer to the average realized prediction accuracy. With respect to the IGC, while our method to compute this index yielded values of 0.49, 0.42, and 0.33 for generations one, five, and ten, respectively, following the derivation based on GBLUP calculated as in Eq. (11), the values obtained were 0.96, 0.86, and 0.75 for generations one, five, and ten, respectively. Finally, these values, if used to compute the curve $\frac{{\left(1+\uprho \right)}^{{\text{r}}}-{\left(1-\uprho \right)}^{{\text{r}}}}{{\left(1+\uprho \right)}^{{\text{r}}}+{\left(1-\uprho \right)}^{{\text{r}}}}$ would result in a great overestimation for ${\text{E}}({\text{R}}|{\text{erosion}})$.

**Fig. 5:**
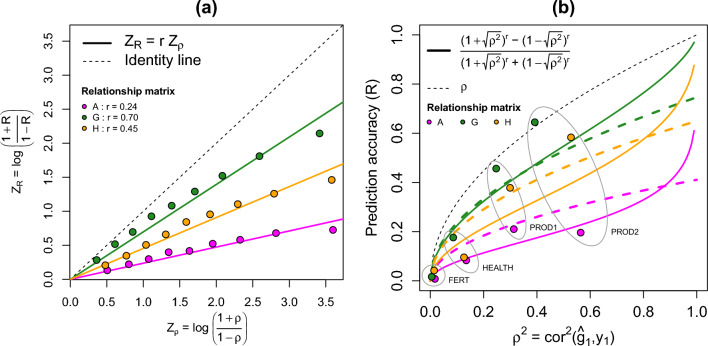
Results of the study on real data: (**a)** Index of genetic correlation (${\text{r}}$) between the reference and target populations using simulated phenotypes and their breeding values solutions as proposed in section IGC from simulated phenotypes. (**b)** Relationship between the realized prediction accuracy ${\text{R}}=\widehat{{\text{cor}}}\left({\widetilde{\mathbf{g}}}_{2},{\mathbf{y}}_{2}\right)$ and the squared accuracy of the breeding values of the reference population, ${\hat {\uprho} }^{2}={\widehat{{\text{cor}}}}^{2}\left({\widehat{\mathbf{g}}}_{1},{\mathbf{y}}_{1}\right)$

Figure 5 and Table 1 present the results on IGC ${\text{r}}$ and on the accuracies of BV and PBV obtained for the reference and target populations, respectively ($\hat {\uprho} =\widehat{{\text{cor}}}\left({\widehat{\mathbf{g}}}_{1},{\mathbf{y}}_{1}\right)$ and ${\text{R}}=\widehat{{\text{cor}}}\left({\widetilde{\mathbf{g}}}_{2},{\mathbf{y}}_{2}\right)$, respectively), for the study on real data and for the four traits evaluated. Figure 5a, for ${\text{r}}$ obtained using the simulated phenotypes, indicates that the pedigree relationship matrix ($\mathbf{A}$) is the matrix that minimizes ${\text{r}}$, while the genomic relationship matrix ($\mathbf{G}$) is the matrix that maximizes ${\text{r}}$. The values of the IGC were ${{\text{r}}}_{{\text{A}}}=0.237$, ${{\text{r}}}_{{\text{G}}}=0.697$, and ${{\text{r}}}_{{\text{H}}}=0.454$, and please recall that the single-step relationship matrix ($\mathbf{H}$) was built using genotypes for all animals in the target population and for 25% of the animals in the reference population. Therefore, the accuracies of the PBV are expected to be lowest when using $\mathbf{A}$, and highest when using $\mathbf{G}$. In fact, we observe in Fig. 5b that accuracies of the PBV are lowest when using $\mathbf{A}$ for all traits, and highest when using $\mathbf{G}$ for most of the traits, except for FERT. However, the 95% confidence intervals (CI) presented in Table 1, for the accuracies of the PBV obtained for FERT, the accuracy obtained when using $\mathbf{G}$ cannot be deemed as significantly different from those obtained when using $\mathbf{A}$ or $\mathbf{H}$. When using $\mathbf{H}$, the results for both ${\text{r}}$ and the accuracies of the PBV are between those obtained when using $\mathbf{A}$ and $\mathbf{G}$. This is not surprising, as the single-step combines the genotype information with the pedigree information from non-genotyped individuals. The values of ${\text{R}}$ obtained when using $\mathbf{A}$ are all close to the curve of their expected values given erosion, i.e. ${\text{E}}({\text{R}}|{\text{erosion}})$, when we observe Fig. 5b; the 95% CI presented in Table 1 for these two values confirm that the observed ${\text{R}}$ are not significantly different from their expectations for all traits. In Fig. 5b, the values of ${\text{R}}$ obtained when using $\mathbf{G}$ appear to be greater than ${\text{E}}({\text{R}}|{\text{erosion}})$; however, the 95% CI presented in Table 1 for these two values indicate that the observed ${\text{R}}$ is significantly different from the expectations only for PROD2. The same conclusions from ${\text{R}}$ obtained when using $\mathbf{G}$ are drawn for the values of ${\text{R}}$ obtained when using $\mathbf{H}$.

**Table 1:** Results for single-trait evaluations performed on real data

Trait	${{\text{h}}}^{2}$	$\sqrt{{{\text{h}}}^{2}}$	Matrix	$\uprho =\widehat{{\text{cor}}}\left({\widehat{{\text{g}}}}_{1},{{\text{y}}}_{1}\right)$		${\text{R}}=\widehat{{\text{cor}}}\left({\widetilde{{\text{g}}}}_{2},{{\text{y}}}_{2}\right)$		$\widehat{{\text{E}}}({\text{R}}|{\text{erosion}})$	
FERT	0.020	0.141	$\mathbf{A}$	0.132	[0.104;0.159]	0.008	[− 0.052;0.068]	0.031	[− 0.029;0.091]
			$\mathbf{G}$	0.073	[0.045;0.101]	0.016	[− 0.044;0.076]	0.051	[− 0.009;0.111]
			$\mathbf{H}$	0.120	[0.092;0.148]	0.042	[− 0.018;0.102]	0.055	[− 0.005;0.114]
HEALTH	0.188	0.434	$\mathbf{A}$	0.367	[0.343;0.391]	0.083	[0.023;0.143]	0.091	[0.031;0.150]
			$\mathbf{G}$	0.294	[0.268;0.319]	0.176	[0.117;0.234]	0.208	[0.150;0.265]
			$\mathbf{H}$	0.356	[0.331;0.380]	0.096	[0.036;0.155]	0.167	[0.108;0.225]
PROD1	0.375	0.612	$\mathbf{A}$	0.560	[0.540;0.597]	0.210	[0.152;0.267]	0.149	[0.090;0.207]
			$\mathbf{G}$	0.497	[0.476;0.518]	0.456	[0.407;0.503]	0.363	[0.310;0.414]
			$\mathbf{H}$	0.547	[0.527;0.567]	0.378	[0.325;0.428]	0.272	[0.215;0.327]
PROD2	0.625	0.791	$\mathbf{A}$	0.751	[0.739;0.763]	0.196	[0.137;0.253]	0.227	[0.207;0.283]
			$\mathbf{G}$	0.626	[0.609;0.643]	*0.654	[0.608;0.679]	0.472	[0.414;0.517]
			$\mathbf{H}$	0.727	[0.713;0.740]	*0.583	[0.542;0.622]	0.396	[0.327;0.445]

^*^Observed ${\text{R}}$ is significantly different from $\widehat{{\text{E}}}({\text{R}}|{\text{erosion}})$, at a significance level of 0.05

Heritabilities (${{\text{h}}}^{2}$) were estimated in a preliminary study, and used to obtain the BV and PBV, which were then used to calculate the accuracies for the reference and target populations: $\hat {\uprho} =\widehat{{\text{cor}}}\left({\widehat{\mathbf{g}}}_{1},{\mathbf{y}}_{1}\right)$ and ${\text{R}}=\widehat{{\text{cor}}}\left({\widetilde{\mathbf{g}}}_{2},{\mathbf{y}}_{2}\right)$, respectively

$\widehat{{\text{E}}}({\text{R}}|{\text{erosion}})$ were calculated according to the indexes of genetic correlation obtained for each of the relationship matrices used for the evaluation, their values being ${{\text{r}}}_{{\text{A}}}=0.237$, ${{\text{r}}}_{{\text{G}}}=0.697$, ${{\text{r}}}_{{\text{H}}}=0.454$. Values between brackets are the 95% confidence intervals for the values presented

## Discussion

We hypothesized that once an IGC between reference and target populations (${\text{r}}$) is calculated, we can define the maximum accuracy of the PBV as the expectation ${\text{E}}\left[{\text{R}}|{\text{erosion}}\right]\le \frac{{\left(1+\uprho \right)}^{{\text{r}}}-{\left(1-\uprho \right)}^{{\text{r}}}}{{\left(1+\uprho \right)}^{{\text{r}}}+{\left(1-\uprho \right)}^{{\text{r}}}}$, such that $\uprho$ represents the true maximum prediction accuracy. We assumed this maximum prediction accuracy to be $\uprho ={\text{cor}}\left({\widehat{\mathbf{g}}}_{1},{\mathbf{y}}_{1}\right)$, the accuracy of the BV obtained for the reference population, as it is intuitive that the accuracy of prediction for the target population cannot be higher than that for the reference population. For ideal scenarios, in which the reference population is sufficiently large, and SNP-genotypes are available and in strong LD with the QTL, we can assume $\uprho \approx \sqrt{{{\text{h}}}^{2}}$, i.e. the theoretical maximum prediction accuracy without erosion. The results obtained with extensive simulations supported our hypothesis, and indicated that indeed considering $\uprho ={\text{min}}\left\{{\text{cor}}\left({\widehat{\mathbf{g}}}_{1},{\mathbf{y}}_{1}\right),\sqrt{{\widehat{{\text{h}}}}^{2}}\right\}$ rather than $\uprho =\sqrt{{{\text{h}}}^{2}}$ resulted in a more correct and consistent ${\text{E}}\left[{\text{R}}|{\text{erosion}}\right]$.

One important element for calculating ${\text{E}}\left[{\text{R}}|{\text{erosion}}\right]$ is the IGC, a single value capable of summarizing all the information about the genetic distance between reference and target populations. When working with genomic prediction, ${\text{r}}$ summarizes the differences in allele frequencies and LD patterns observed in both the reference and target populations. We presented two methods to calculate ${\text{r}}$, one method that simulates phenotypes and predicts them on the target population to infer ${\text{r}}$, and another method that can only be applied for genomic prediction by performing operations on the SVD of the genotype matrices from the reference and target populations. Our results show that both methods to obtain ${\text{r}}$ are trustworthy.

Although computationally costly for large populations and dense genotype data, calculating ${\text{r}}$ using the SVD of the genotype matrices may offer extra information about the genetic similarities or differences of the populations. Such decompositions are a very informative tool to evaluate the connections between the individuals studied, and the LD between the SNPs. Then, it is intuitive that allele frequencies, LD patterns, number of SNPs and population sizes affect the IGC by changing the coefficients ${\text{a}}$, ${\text{b}}$, and ${\text{c}}$ that compose ${\text{r}}={\text{a}}+{\text{b}}+{\text{c}}$ (a result obtained based on the extensive observations of empirical results). Because this work focused on calculating ${\text{E}}\left[{\text{R}}|{\text{erosion}}\right]$, we did not explore the underlying meaning of the values of these coefficients (i.e. how they are affected by allele frequencies, LD patterns, number of SNPs and population sizes), but we do understand that such a study may be relevant, and should be conducted in the future.

When we compared the theoretical curves of ${\text{E}}\left[{\text{R}}|{\text{erosion}}\right]$ obtained for the different relationship matrices ($\mathbf{A}$, $\mathbf{G}$, and $\mathbf{H}$) to the realized prediction accuracies, we observed that the curve for $\mathbf{A}$ was the curve that best outlined expectations for the prediction accuracies using this relationship matrix. The realized prediction accuracies obtained using $\mathbf{G}$ and $\mathbf{H}$ were farther from the theoretical curves of ${\text{E}}\left[{\text{R}}|{\text{erosion}}\right]$, however only the realized prediction accuracies for the PROD2 trait were significantly different from the expectations, as shown in Table 1.

PROD2 was the trait with the highest heritability (${{\text{h}}}^{2}=0.625$). However, Fig. 4b, which presents the results on simulated data, indicated that the variance of prediction accuracies increases with the heritability of the trait. Thus, the variance of the prediction accuracy for PROD2 should be larger than the variances of the prediction accuracy for the other three traits. Although the realized prediction accuracies of PROD2 were significantly higher than ${\text{E}}\left[{\text{R}}|{\text{erosion}}\right]$, we have to look at this result with caution. The CI presented in Table 1 are for correlations, and rely on the Z-transformed correlation, which are estimates, rather than exact CI. Moreover, it is relevant to consider that the real data evaluated comprised phenotypes measured on a breeding population, i.e. under selection, meaning that this may increase the accuracy of PBV obtained with genomic data, as selection can lead to an increase in the LD between the QTL and the most relevant SNPs, resulting in more accurate estimates for the SNP effects in the later populations. Thus, one possible explanation for the observed prediction accuracies of PROD2 being significantly higher than ${\text{E}}\left[{\text{R}}|{\text{erosion}}\right]$ may be that, due to selection favouring a highly heritable trait, a stronger LD between the most relevant SNPs and the QTL is present, increasing the relationships between reference and target populations at the SNPs with a larger effect. Thus, genomic PBV for young candidates in a breeding program are expected to be quite accurate for highly heritable traits, even if the IGC between reference and target populations is low because the most significant SNPs have their effects quite accurately estimated in the reference population, and moreover, due to selection, reference and target populations should not present large differences for those SNPs with larger effects. Thus, if the SNPs that drive low values of ${\text{r}}$ are those that are less significant, then ${\text{r}}$ will have a smaller relevance for ${\text{E}}\left[{\text{R}}|{\text{erosion}}\right]$. We note that the observed prediction accuracies of PROD2 were also greater than those of ${\text{E}}\left[{\text{R}}|{\text{GBLUP}}\right]$.

The accuracy of PBV has been previously studied from different perspectives [6, 17, 27, 28], and different deterministic equations have been proposed to calculate this accuracy [6, 15, 16, 18, 19, 29, 30]. The degrees of the genomic relationships [27, 28], as well as the LD and co-segregation of the QTL from pedigree [17], have already been evaluated as contributors to the accuracy of genomic prediction.

In order to predict the accuracy of genomic PBV, Goddard et al. [30] derived a method using the total genetic variance and the pairwise genomic correlations between individuals. Using a different approach, Wientjes et al. [18, 19] proposed a deterministic equation to predict the accuracy of PBV, accounting for differences between populations in across-breed predictions, and ${{\text{M}}}_{{\text{e}}}$, a function of the genotype data. Lee et al. [21] extended the proposed equation from Wientjes et al. [18, 19] by accounting for the effective population size (${{\text{N}}}_{{\text{e}}}$) and studied how the degrees of the relationships between individuals, the size of the reference population and marker panel density impact the prediction accuracy. On the one hand, our study with simulated data, shows that the aforementioned deterministic methods yielded predictions for the accuracy of the genomic PBV that were very similar to the curves of ${\text{E}}\left[{\text{R}}|{\text{GBLUP}}\right]$. Also using a deterministic approach, Dekkers et al. [15] used selection index theory and Fisher’s information theory to predict the accuracy of PBV, a method that ultimately relies on the information about the erosion at the individual SNP level. However, accurately quantifying the erosion at the individual SNP level is a difficult and unresolved task. On the other hand, our work shows that quantifying the erosion of the accuracy of the PBV as a population parameter is a more tractable problem.

Taking a different approach from what was previously proposed, we defined a metric to quantify the IGC between reference and target populations. Then, we used this correlation to derive a statistical prediction for the accuracy of PBV, ${\text{E}}\left[{\text{R}}|{\text{erosion}}\right]$, based on Fisher’s Z-transformation [23] and treating the accuracy of the PBV as a population parameter, and demonstrated through simulated and real data that our derived ${\text{E}}\left[{\text{R}}|{\text{erosion}}\right]$ is a reliable metric.

## Conclusions

The accuracy of PBV is a very important factor for the success of breeding programs that make use of estimates of genetic merit to select individuals. While the advent of genomic prediction has greatly increased the accuracy of PBV, realized accuracies remain below $\sqrt{{{\text{h}}}^{2}}$, even when the reference population is sufficiently large, and SNPs included in the model are in sufficient LD with the QTL. This is particularly noticeable across generations, as we observe the so-called erosion of SNP effects [15] accompanied by the erosion of the accuracy of the PBV. We defined an IGC between reference and target populations, which summarizes the expected overall erosion due to differences in allele frequencies and LD patterns between reference and target populations. We used this correlation to derive a statistical prediction for the accuracy of the PBV accounting for erosion, ${\text{E}}\left[{\text{R}}|{\text{erosion}}\right]$, an expectation based on Fisher’s Z-transformation, and demonstrated that our derived ${\text{E}}\left[{\text{R}}|{\text{erosion}}\right]$ is a reliable metric.

### Supplementary Information

**Additional file 1.** Example_IGC. Sample code to simulate genotypes/phenotypes, perform genomic prediction and compute the index of genetic correlation (IGC) between reference and target populations.

## Data Availability

Simulations of genotypes and phenotypes were performed using the R [31] package GenEval (https://github.com/bcuyabano/GenEval/). A sample code for implementing the method in R is provided in Additional file 1. The real data analysed are from a commercial source and not publicly available.
